# Optimizing experimental designs for model selection of ion channel drug-binding mechanisms

**DOI:** 10.1098/rsta.2024.0227

**Published:** 2025-03-13

**Authors:** Frankie Patten-Elliott, Chon Lok Lei, Simon P. Preston, Richard D. Wilkinson, Gary R. Mirams

**Affiliations:** ^1^ Centre for Mathematical Medicine & Biology, School of Mathematical Sciences, University of Nottingham, Nottingham, UK; ^2^ Faculty of Health Sciences, Institute of Translational Medicine, University of Macau, Macau, People’s Republic of China; ^3^ Department of Biomedical Sciences, Faculty of Health Sciences, University of Macau, Macau, People’s Republic of China

**Keywords:** hERG, mathematical model, safety pharmacology, binding mechanism, model discrepancy, experimental design

## Abstract

The rapid delayed rectifier current carried by the human Ether-à-go-go-Related Gene (hERG) channel is susceptible to drug-induced reduction, which can lead to an increased risk of cardiac arrhythmia. Establishing the mechanism by which a specific drug compound binds to hERG can help reduce uncertainty when quantifying pro-arrhythmic risk. In this study, we introduce a methodology for optimizing experimental voltage protocols to produce data that enable different proposed models for the drug-binding mechanism to be distinguished. We demonstrate the performance of this methodology via a synthetic data study. If the underlying model of hERG current is known exactly, then the optimized protocols generated show noticeable improvements in our ability to select the true model when compared with a simple protocol used in previous studies. However, if the model is not known exactly, and we assume a discrepancy between the data-generating hERG model and the hERG model used in fitting the models, then the optimized protocols become less effective in determining the ‘true’ binding dynamics. While the introduced methodology shows promise, we must be careful to ensure that, if applied to a real data study, we have a well-calibrated model of hERG current gating.

This article is part of the theme issue ‘Uncertainty quantification for healthcare and biological systems (Part 1)’.

## 1. Introduction

Ion channels are proteins in the cell membrane that form pores through which ions can flow in and out of the cell. The resulting ion currents play an important role in several biological functions including coordinating the contraction of muscle cells. A healthy heart relies on regular, coordinated contractions of cardiomyocytes (heart muscle cells) to pump blood from the heart around the body [1]. The *K*_v_11.1 ion channel encoded by the human Ether-à-go-go-Related Gene (hERG) is responsible for conducting the rapid delayed rectifier potassium current, $I_{\text{Kr}}$, and plays a crucial role in cardiomyocytes recovering from excitation [2]. However, the hERG channel is susceptible to unintended block by pharmaceutical small molecules (referred to here as ‘compounds’ throughout); this can lead to a reduction in $I_{\text{Kr}}$, lengthening the cardiac action potential, and, in some cases, increasing the risk of cardiac arrhythmia [3–5].

Markov-style computational models of ion channels define transition rates between several channel states (e.g. open, inactive and closed) and can be used to simulate channel current in response to a membrane potential. To model the interactions of drug compounds with an ion channel, additional states and rates can be introduced to an existing ion channel model to simulate various binding mechanisms [6]. When it comes to the hERG channel, it has been observed that the binding mechanism can be compound-specific [7–9]. One such example of this is the propensity of a compound to become ‘trapped’ (unable to unbind) when the channel closes. Some compounds, such as bepridil and dofetilide, are known to become trapped inside the central hERG cavity, remaining bound, while others, such as cisapride and verapamil, unbind when the channel closes [10–12]. It has also been theorized that some drugs bind preferentially to certain channel states over others, or in the extreme case, bind to a particular state only [13–17]. These compound-specific binding mechanisms suggest that a one-size-fits-all approach to modelling hERG-drug interactions is perhaps limiting [6]. To accurately model how a certain compound binds to the hERG channel, it is, therefore, important to determine the specific mechanisms at play.

The transmembrane current of a cell can be measured in response to the membrane potential via a voltage-clamp experiment, where a piecewise function defining the transmembrane voltage, V, dependent on time, t, is applied. We refer to this function, V(t), as a *voltage protocol*. In a recent study, Lei *et al*. [18] considered fitting a set of 15 pharmacological models representing possible drug-binding mechanisms (trapping/non-trapping, state binding preference, etc.) to previously collected voltage-clamp data under a relatively simple protocol. After fitting the set of models, it was suggested that more information-rich experiments may be needed to distinguish between model outputs and assist in determining compound-specific binding mechanisms. Previous studies on drug-binding dynamics have considered ‘manual’ experimental design techniques to increase the information extracted from voltage-clamp experiments [19–23]. This often involves some degree of expert knowledge to design protocols that are expected to emphasize particular compound-specific behaviours. In this work, we instead consider ‘automated’ optimal experimental design (OED) techniques.

OED methods consider how the design of a data-collecting experiment can be optimized with respect to some statistical criterion, effectively maximizing the information provided by the experiment (subject to constraints). These methods have been used recently in the field of cardiac modelling with some success [24]. In this paper, we consider OED methods to design voltage protocols that can be used in voltage-clamp experiments to better distinguish between different models of drug-binding mechanism. We detail a synthetic data methodology for generating an optimized protocol and fitting models to data collected under this protocol. Our results demonstrate how the optimized designs can assist in establishing the true binding dynamics at play across a range of simulated drug compounds exhibiting differing dynamics. However, we find that introducing a *discrepancy* between the hidden ‘true’ data-generating model and the proposed model we work with to fit the data reduces the effectiveness of this method and suggests that further work may be needed to account for these inevitable model discrepancies when working with real data.

## 2. Mathematical models

### (a) hERG physiological models

**Figure 1.**
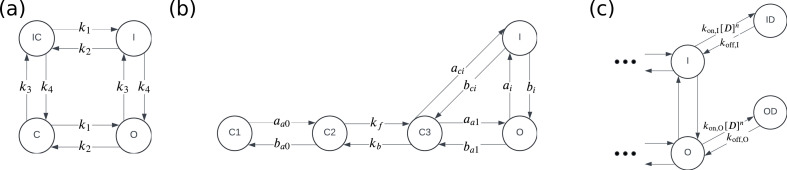
Markov diagrams of physiological hERG models (a,b) and a pharmacological binding model (c). The model in (a) is a four-state symmetric hERG model as used by Lei *et al.* [18]. The four states are IC (inactive closed), C (closed), I (inactive) and O (open). The model in (b) is a five-state model equivalent to that derived by Lu *et al.* [25]. This model has three closed states (C1, C2 and C3) as well as O (open) and I (inactive) states. The pharmacological drug-binding in (c) is Model 7 from Lei *et al.* [18]. The I and O states represent the inactive and open states in the underlying physiological hERG models (this model could be attached to the right-hand side of either physiological model shown in (a) or (b)). The binding model additionally has two drug-bound states ID (inactive drug-bound) and OD (open drug-bound), in which no current flows.


In this paper, we consider two physiological models of hERG; one simple four-state model that is used throughout and a slightly more complex five-state model exhibiting differing behaviour that is used when we introduce hERG model discrepancy. These models describe the voltage-dependent gating behaviour of $I_{\text{Kr}}$ at physiological temperature when no drug compound is present. Figure 1 shows Markov diagrams of the two hERG models we consider. Figure 1a shows the four-state model, with transition rates $k_{1}$–$k_{4}$, which is equivalent to physiological model B in Lei *et al.* [18]. The four states in this model are IC (inactive closed), C (closed), I (inactive) and O (open), where each variable represents the proportion of channels in that state. Figure 1b shows the five-state model from Lu *et al.* [25], with transition rates $a_{a0}$, $b_{a0}$, $k_{f}$, $k_{b}$, $a_{a1}$, $b_{a1}$, $a_{ci}$, $b_{ci}$, $a_{i}$ and $b_{i}$. This model has three closed states (C1, C2 and C3), and no states that are both inactive and closed. All transition rates in both models (apart from $k_{f}$ and $k_{b}$) are dependent on transmembrane voltage, V; they are defined by the general equation $p_{i}\text{exp}(p_{j}V)$ where $p_{i}$ and $p_{j}$ are physiological model parameters taken from the literature. In both models, the rate of change of each state over time, t, is defined by a differential equation. For example, for the model in figure 1a, the rate of change of open proportion is defined by

(2.1) $$\begin{array}{lrr} & \frac{d\text{O}}{dt}=k_{1}\text{C}+k_{4}\text{I}-(k_{2}+k_{3})\text{O}. & \end{array}$$

The measured current, $I_{\text{Kr}}$, is then calculated for both models via the following equation


(2.2) $$\begin{array}{lrr} & I_{\text{Kr}}=g_{\text{Kr}}\cdot \text{O}\cdot (V-E_{K}), & \end{array}$$

where $g_{\text{Kr}}$ is conductance, O is the open state proportion and $E_{K}$ is the Nernst potential (membrane potential at which there is no net flux).

### (b) Pharmacological binding models for hERG

**Figure 2.**
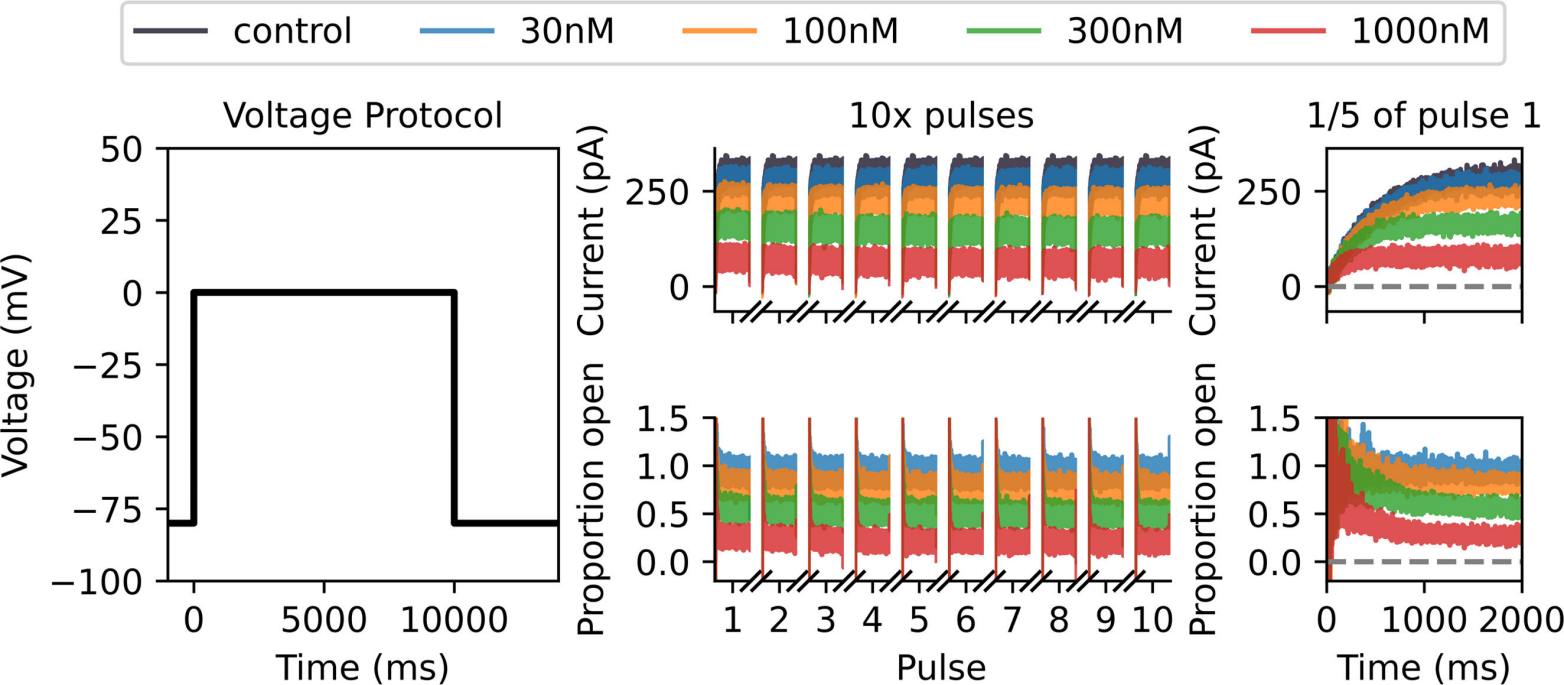
Synthetic verapamil data under drug-binding Model 7 generated under a Milnes protocol with the Lei 37°C hERG model. On the left, we plot a single sweep of the Milnes protocol. In the top middle, we plot the control (in black) and drug currents (blue, orange, green and red corresponding to four different drug concentrations) for 10 sweeps of the Milnes protocol. We only plot the currents that occur during the 10 s pulse at 0 mV for each sweep. In the bottom middle, we plot the corresponding proportion open for each drug concentration, which is calculated by dividing each drug sweep by the control current. On the right-hand side, we include a zoomed-in look at the first $\frac{1}{5}$ (2 s) of the first pulse for both currents and proportion open. This illustrates the currents starting at approximately 0 pA and the open proportion starting at approximately 1. The noise in the initial low currents contributes to the increased noise at the beginning of the open proportion sweeps. This motivates using a noise model for the ratio of two normally distributed variables when fitting drug-binding models to this data.

We can extend the Markov models of hERG described in the previous section to model drug-binding dynamics in hERG. We consider a set of 15 models that characterize different proposed candidate mechanisms for drug-binding as illustrated in Lei *et al.* ([18]; figure 2) and included in our electronic supplementary material, fig. S1. Figure 1c shows a Markov diagram of drug-binding Model 7 as an example of one of these 15 models. In this example model, we have two additional states: ID (inactive drug-bound) and OD (open drug-bound). Binding rates are described by the parameters $k_{\text{on,I}}$, $k_{\text{off,I}}$, $k_{\text{on,O}}$ and $k_{\text{off,O}}$, and the rate of on-binding is dependent on the drug concentration [D] and the Hill coefficient n. This example model represents *non-trapping* binding behaviour as the channel cannot enter a closed state without the drug first unbinding (returning from an ID or OD state to an I or O state). In contrast, some of the 15 binding models represent *trapping* dynamics. The trapping component either involves a ‘mirror image’ of the physiological hERG model, which allows for a channel to close and prevent unbinding from ICD or CD states (corresponding to IC and C in the drug-bound channel) or is represented by additional trapped states. In electronic supplementary material, fig. S1, Models 4, 5, 5i, 6, 9 and 10 illustrate the ‘mirror image’ trapping, while Models 11, 12 and 13 include additional trapped states.

## 3. Introducing the OED methodology

### (a) Initial synthetic data

To measure the effect of drug block on the hERG channel, we can collect two sets of voltage-clamp time-series data; the control current, y(t), before the drug compound is introduced and the current in the presence of the drug compound, $x_{c}(t)$ (often at several different concentrations c∈C). We begin by generating synthetic ion channel drug-binding data in a form resembling what we would expect to collect in a voltage-clamp experiment under a simple modified Milnes voltage protocol as used in the Comprehensive *in vitro* Proarrhythmia Assay initiative [19,26]. With these synthetic data, we fit the set of 15 drug-binding models from electronic supplementary material, fig. S1 and illustrate a need for a more complex protocol to differentiate between these models. The process by which these synthetic data is generated is described as follows. All electrophysiology simulations are performed in Myokit [27].

In figure 2, we plot the Milnes protocol, i.e. controlled voltage time-series (left), synthetic current data (top middle) and synthetic open proportion data (bottom middle). In the synthetic current plot, the control current in black, y(t), is generated under the Lei Markov model described in figure 1a with parameters for physiological temperatures taken from Lei *et al.* [28]. We run 10 sweeps (repeats in series) of the Milnes protocol and plot the 10 s of each sweep corresponding to the 0 mV pulse in the protocol. We generate equivalent currents in the presence of four concentrations of verapamil ($x_{c}(t),c\in C=\left\{30,100,300,1000\right\}$), and these are plotted in blue (30 nM), orange (100 nM), green (300 nM) and red (1000 nM). Gaussian random noise is added to all sweeps with a standard deviation of 10 pA. We use a step size of 0.5 ms between data points, and we can define T as the set of all times for the plotted traces. The plotted proportion of channels open, $z_{c}(t)$, in the bottom middle of figure 2 is calculated by dividing the drug current sweeps, $x_{c}(t)$, by the control sweep, y(t), and this is the normalized quantity we use to fit the drug-binding models, as in [26,29].

As an example for many of the figures in this paper, for our true data-generating model of drug-binding dynamics, we have used the drug-binding Model 7 described in Lei *et al.* [18] with parameters estimated from verapamil voltage-clamp data collected at physiological temperatures by Li *et al.* [26,29]. Lei *et al.* [18] found that this model gave ‘plausible’ fits to the Li *et al.* data and agrees with the literature that verapamil does not tend to become trapped. This non-trapping behaviour involves having no bound closed state in the binding model; the drug can only be bound and block the channel when the channel is in an OD or ID state. In §4, we go on to examine findings if any other binding model was the true data-generating model for other drugs as well.

### (b) Initial model fitting

Next, we fit each of the 15 drug-binding models to the synthetic ‘proportion open’ data, $z_{c}(t)$. As a first pass, we assume that the underlying Lei hERG model is known exactly (i.e. there is no hERG model discrepancy and the model parameters are known) and we wish to fit only the parameters of the drug-binding models. The data, $z_{c}(t)$, are generated by dividing two current traces which both have normally distributed iid noise, i.e. the data are a ratio of two normally distributed random variables. If $X∼N(\mu_{X},\sigma_{X}^{2})$ and $Y∼N(\mu_{Y},\sigma_{Y}^{2})$ are two independent normal random variables, then the ratio Z=X/Y has probability density function (PDF) given by [30]


(3.1) (9)fZ(z;β,ρ,δ)=ρπ(1+ρ2z2)(exp{−ρ2β2+12δ2}+qπ2erf(q2)exp{−ρ2(z−β)22δ2(1+ρ2z2)}),

where $\beta =\frac{\mu_{X}}{\mu_{Y}}$, $\rho =\frac{\sigma_{Y}}{\sigma_{X}}$, $\delta =\frac{\sigma_{Y}}{\mu_{Y}}$ and


(3.2) $$\begin{array}{lrr} & q=\frac{1+\beta \rho^{2}z}{\delta \sqrt{1+\rho^{2}z^{2}}}. & \end{array}$$

For our synthetic $z_{c}(t)$ data, $\mu_{X}(t,c,\theta )$ is the modelled current at time t in the presence of a drug compound of concentration c under some drug-binding parameterization θ, while $\mu_{Y}(t)$ is the control current at time t and does not depend on θ or c. We simplify things by assuming that $\sigma_{X}$ and $\sigma_{Y}$, the standard deviations of the measurement error on the drug and control currents, respectively, are equal ($\sigma_{X}=\sigma_{Y}=\sigma$) resulting in ρ=1. We can then use the PDF in equation (3.1) to derive a log-likelihood function for some drug-binding model parameterization θ and standard deviation σ given a dataset z


(3.3) $$\begin{array}{r} L(\theta ,\sigma |z)=\sum_{c\in C}\;\sum_{t\in T}\text{log}\;f_{Z}(z_{c}(t);\beta (t,c,\theta ),1,\delta (t,\sigma )). \end{array}$$

Each of our 15 drug-binding models can then be fitted to $z_{c}(t)$ by maximizing this log-likelihood function with respect to θ and σ.

**Figure 3.**
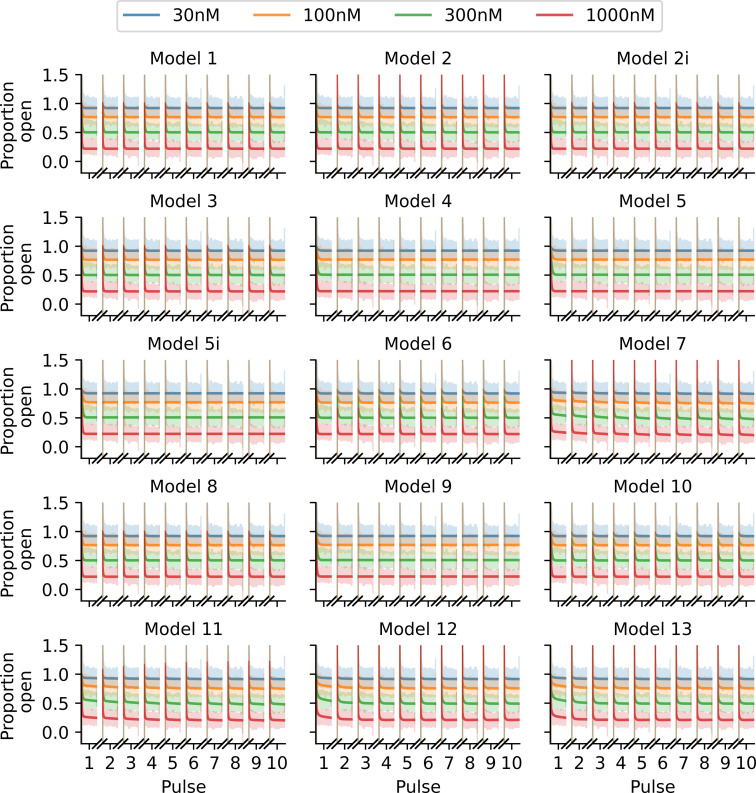
Fits of the 15 binding models to the Milnes protocol proportion-open synthetic data shown in figure 2. Note that it is difficult to visually distinguish between the quality of the model fits for this protocol. The fitted models are shown with solid lines, and we plot the data shaded behind these fits.

We use the covariance matrix adaptation evolution strategy (CMA-ES) optimization algorithm [31] via the probabilistic inference on noisy time-series framework [32] to perform this maximization. We repeat the CMA-ES optimization 10 times, with each repeat starting from a different parameter initialization sampled from wide boundaries as described in [18], and take the largest obtained log-likelihood. The choice of 10 repeats is motivated by a desire to balance computation time with accuracy; on average, 8 of the 10 repeats give very similar maximized likelihoods and corresponding parameter estimates. In figure 3, we plot the fits obtained via this method for each of the 15 drug-binding models. It is difficult to visually distinguish between the quality of these fits and, at a glance, it appears that all models fit the data relatively well.

**Figure 4.**
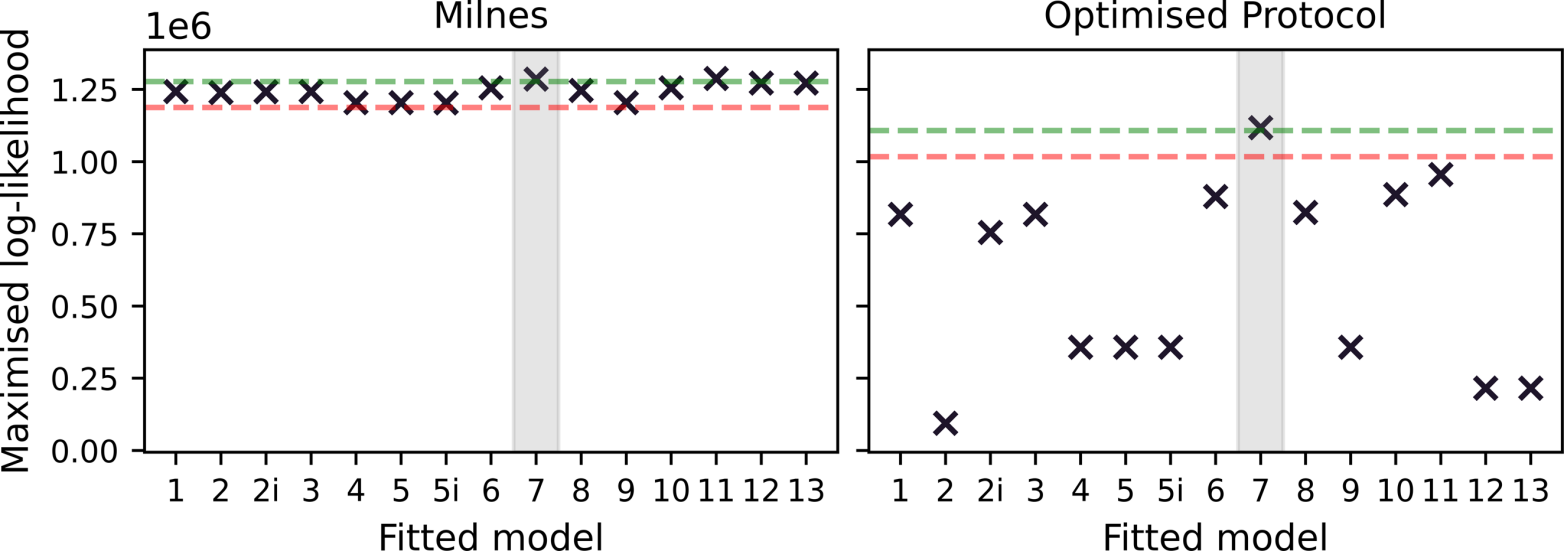
Maximized log-likelihoods for model fits to synthetic data generated under the modified Milnes protocol (left) and an optimized protocol (right). In each plot, we include a green dashed line at $10^{4}$ below the largest maximized log-likelihood and a red dashed line at $10^{5}$ below the largest maximized log-likelihood. We note that these lines act simply as visual aids to emphasize the increased spread in the quality of model fits under the optimized protocol. We also shade, in grey, around the true data-generating binding model.

In the left-hand plot of figure 4, we plot the maximized log-likelihood values obtained when fitting each model under the Milnes protocol. The true data-generating model (Model 7) and one with a very similar structure (Model 11) have the largest maximized log-likelihoods, while all other models fall within a range of $10^{5}$ below this value. We note that we are fitting each model to very high-resolution data ($8\times 10^{4}$ data points), which gives rise to large log-likelihood values. A traditional model selection method such as the likelihood ratio test or the Akaike information criterion (AIC) may suggest, based on the differences in log-likelihoods, that the data-generating model is chosen over the other models. However, in a real data scenario, we generally have less confidence in the veracity of the model of observed hERG current owing to experimental artefacts [33], so we avoid using likelihood ratio testing or AIC in this context. Ultimately, we arrive at the same conclusion drawn by Lei *et al.* [18]; this protocol is not information-rich enough to distinguish between drug-binding models, which motivates our use of OED methods.

### (c) Optimizing the experimental design

Our next step is to develop an improved experimental design that emphasizes differences between drug-binding models. We do this by employing OED techniques. Let us assume that we have some experimental design, D, that is a function of some parameter set ϕ. We then need to establish some optimality criterion, g, that is a function of the design, and determine a ϕ that maximizes g. We begin by considering g; what do we want to optimize? Practically speaking, we want a protocol that accentuates the differences between the models for a specific drug compound. This motivates the following optimality criterion.

#### (i) Optimality criterion

Assume we have our fitted models from figure 3 and these are represented by $\beta_{m}(t,c,{\hat{\theta }}_{m},\phi )$ for m∈M where M is the set of 15 drug-binding models and ${\hat{\theta }}_{m}$ is the maximum likelihood estimate (MLE) parameter set for model m. Note we have included ϕ as an input to $\beta_{m}$ here, as $\beta_{m}$ is dependent on the experimental design. We can then calculate the pairwise squared difference between each of the m model outputs, which we label $d_{ij}$, where


(3.4) $$\begin{array}{r} d_{ij}(\phi )=\sum_{c\in C}\;\sum_{t\in T}{\left(\beta_{i}(t,c,{\hat{\theta }}_{i},\phi )-\beta_{j}(t,c,{\hat{\theta }}_{j},\phi )\right)}^{2}. \end{array}$$

We can then propose that our optimality criterion seeks to maximize the median value of $d_{ij}(\phi )$ across all i,j∈M, i≠j, which we call $d_{med}(\phi )$


(3.5) $$\begin{array}{lrr} & \hat{\phi }=\underset{\phi }{\text{max}}\;d_{med}(\phi ). & \end{array}$$

There are many choices of design criteria to optimize. A conventional option is the T-optimality criterion introduced by Atkinson & Fedorov [34], which involves maximizing the *minimum* pairwise difference. However, in our case with some pairs of very similar models, initial experimentation with a T-optimality approach often resulted in the two most similar models being separated slightly, while the optimizer would ignore the pairwise differences between the other 13 models. Here we favour the *median* to ensure that the objective will seek to increase the pairwise differences between at least half of the 15 models.

#### (ii) Optimizing the protocol

We now turn our attention to D(ϕ). With consideration of experimental practicality, we can establish a design space that we want to optimize the max-med criterion over. We begin by splitting the 10 s 0 mV pulse used in the Milnes protocol into three separate steps (of length 3340, 3330 and 3330 ms) each of which can be set to a voltage in the range of −50 to +40 mV. To increase the design space, we include two 10 s pulses per sweep and allow both pulses to each have three different voltage steps. Further to this, we allow the times between pulses at the holding potential of −80 mV to vary; the time following the first pulse and second pulse can be set to be anywhere between 1050 and 21 000 ms. In total, we then have eight degrees of freedom for optimizing the max-med criterion; six voltage step values and two interpulse durations. Let us then define $\phi =\left[V_{11},V_{12},V_{13},V_{21},V_{22},V_{23},t_{1},t_{2}\right]$ where $V_{ij}$ is the jth voltage step (in mV) in the ith pulse in the protocol and $t_{i}$ is the time (in ms) following the ith pulse in the protocol. We can then optimize the max-med criterion with respect to ϕ via CMA-ES to determine an optimal protocol $D(\hat{\phi })$. To initialize the CMA-ES optimization, we take 100 random samples from within the voltage and time-step bounds defined above ([−50, +40] and [1050, 21 000], respectively), and then use the ϕ that gives the largest value of the max-med criterion as our initialization for the optimizer. We note here that finding the global optimum does not necessarily matter, our goal here is simply to find an improved design.

### (d) Fitting models to synthetic data generated with an optimized protocol

**Figure 5.**
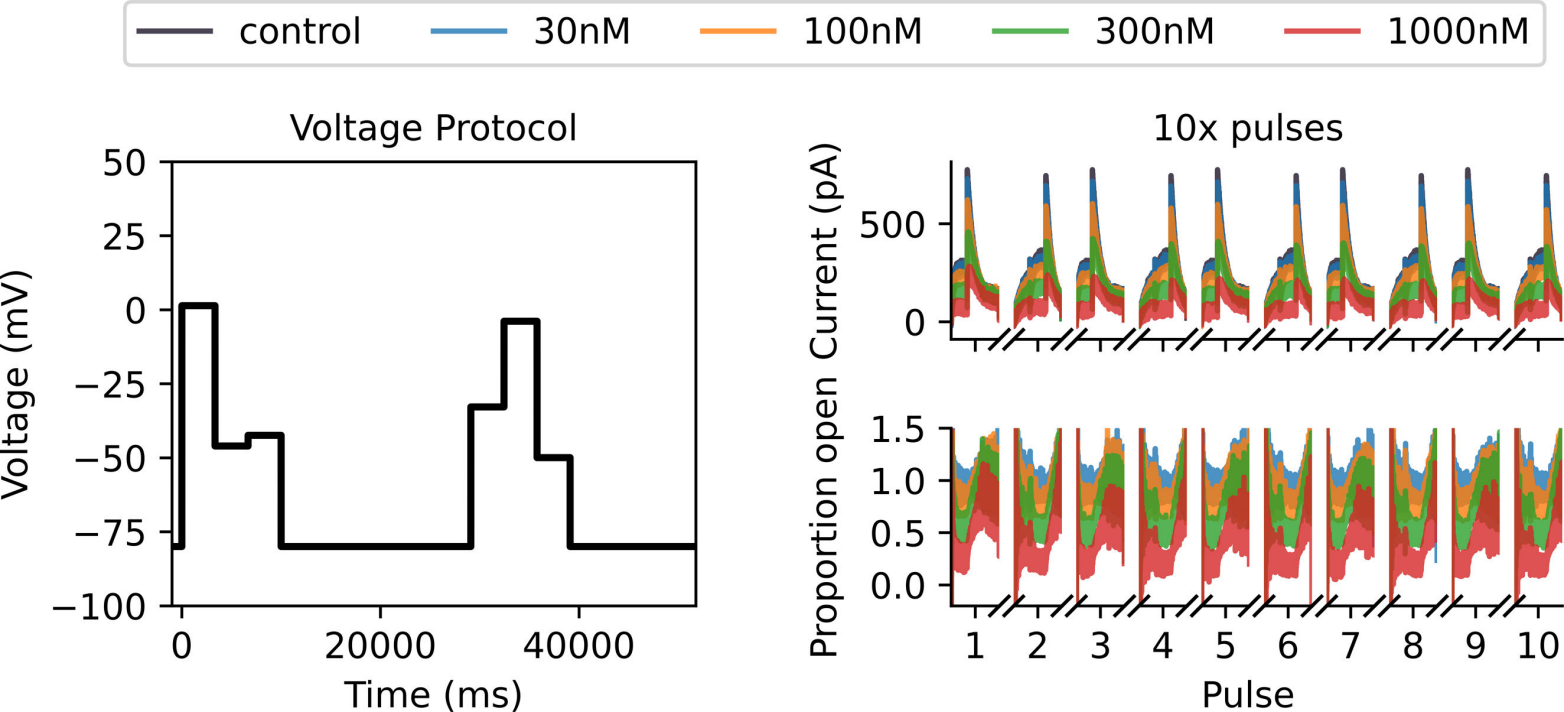
Synthetic data for drug-binding Model 7 generated under an optimized protocol with the Lei 37°C hERG model. On the left-hand side, we plot our optimized protocol with two 10 s pulses, each with three optimized voltage steps. Top right shows the control and drug currents for five sweeps of the optimized protocol. We only plot the currents that occur during the two 10 s pulses per protocol sweep. The bottom right-hand plot shows the corresponding proportion open for each drug concentration which is calculated by dividing each drug sweep by the control current.

**Figure 6.**
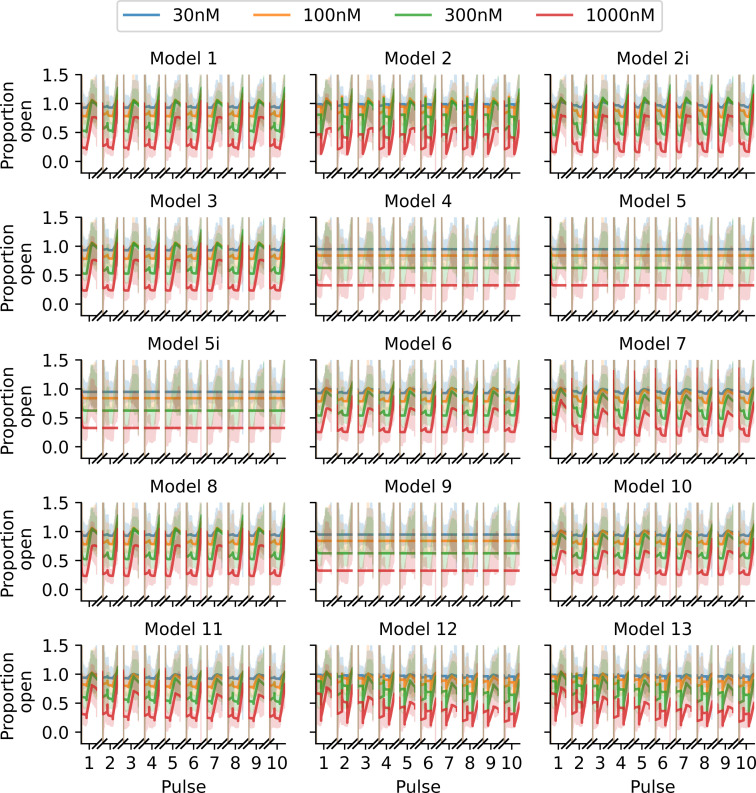
Fits of the binding models to the optimized protocol proportion open synthetic data shown in figure 5, in the same style as figure 3. Note how many fits are now visually distinguishable, we can immediately see that many models provide a worse fit to these data than others.

With an optimized protocol, we can then generate a new set of synthetic data using the methodology described in §3*a*. Our new protocol is comprised of two multistep 10 s pulses of interest, whereas the Milnes protocol has just one single-step 10 s pulse. We, therefore, generate data for only five sweeps (cf. 10) of our new protocol to ensure that the synthetic data under the optimized protocol have the same number of data points as the Milnes synthetic data. In figure 5, we plot this new synthetic current and proportion open data (right top and bottom, respectively) alongside the optimized protocol (left). We can then fit the 15 drug-binding models to this new synthetic data using the same method described in §3*b*; the fits are shown in figure 6. We see that, when compared with figure 3, we now have a number of models that do not appear to fit the data very well. This is backed up by the right-hand plot in figure 4, where we see a greater spread in maximized log-likelihood values in the optimized protocol case compared with the Milnes case, making it easier to distinguish between models. Model 7, the true data-generating model, has the largest maximized log-likelihood; while no other models have maximized log-likelihoods within $10^{5}$ of Model 7. In the electronic supplementary material, fig. S2, we include a plot comparing the model parameters fitted to the Milnes data and the model parameters fitted to the optimized protocol data.

### (e) A discrepant hERG model

So far, we have been working under the assumption that the structure and parameterization of the underlying data-generating hERG model are known exactly. Bernardo & Smith [35] describe this as an M-closed model space, where the true data-generating model is included in the set of models considered for model selection. We can never guarantee this practically, so we now introduce an example where the assumed hERG model used in the model fitting and protocol optimization steps is different from the hERG model used during the data-generating process. We are now operating in an M-open model space where the true data-generating model is not within our set of candidate models.

**Figure 7.**
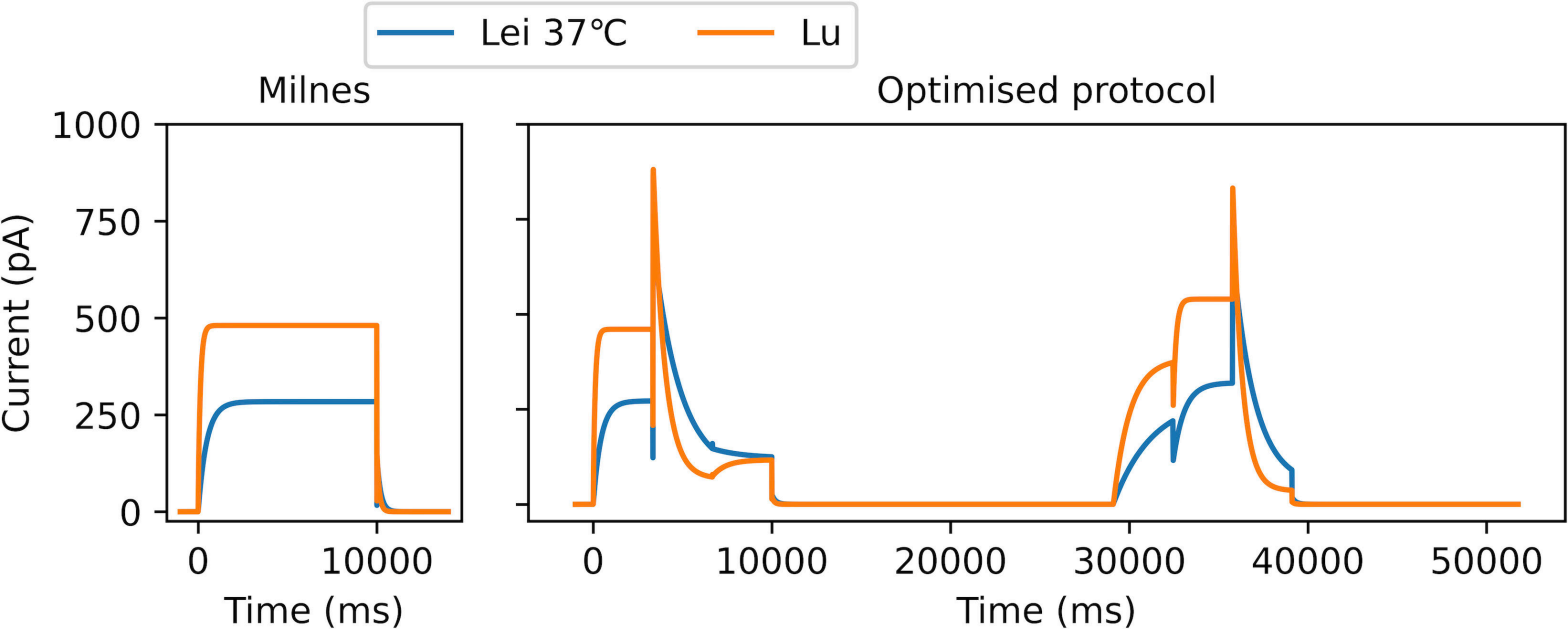
A comparison of control currents between the Lei model (figure 1*a*) parameterized for 37°C and the Lu model (figure 1*b*). To the left, we plot the currents in response to one sweep of the Milnes protocol, and to the right in response to one sweep of the optimized protocol from figure 5. Conductances have been set to 33.3 nS for both models.

To generate synthetic data, we now use the Lu model as illustrated in figure 1*b*. In figure 7, we plot a comparison between control currents under the Lu model and the 37°C Lei model previously used to generate synthetic data. We set the conductances ($g_{\text{Kr}}$) for both models to be equal. We note that the difference in dynamics between these two models appears to be quite significant; for example, there is an approximately 350 ms difference in the time constants of activation, $\tau_{a}$ (measured during the Milnes protocol step to 0 mV). Sanguinetti & Jurkiewicz [36] approximated hERG $\tau_{a}$ as 50 ms based on experimental data collected under approximately similar experimental conditions (a voltage step to 0 mV at 35°C). Comparing this experimental estimate with the $\tau_{a}$ approximated for the Lei and Lu models, we get differences of 400 and 50 ms, respectively. The difference between our model dynamics (at least regarding activation times) falls within this range of differences between models and real data. While this model difference is potentially on the larger end of what we would want from a well-calibrated model of hERG when compared with real data, we consider this a stress test of how our OED methodology performs when there is a relatively large model discrepancy.

**Figure 8.**
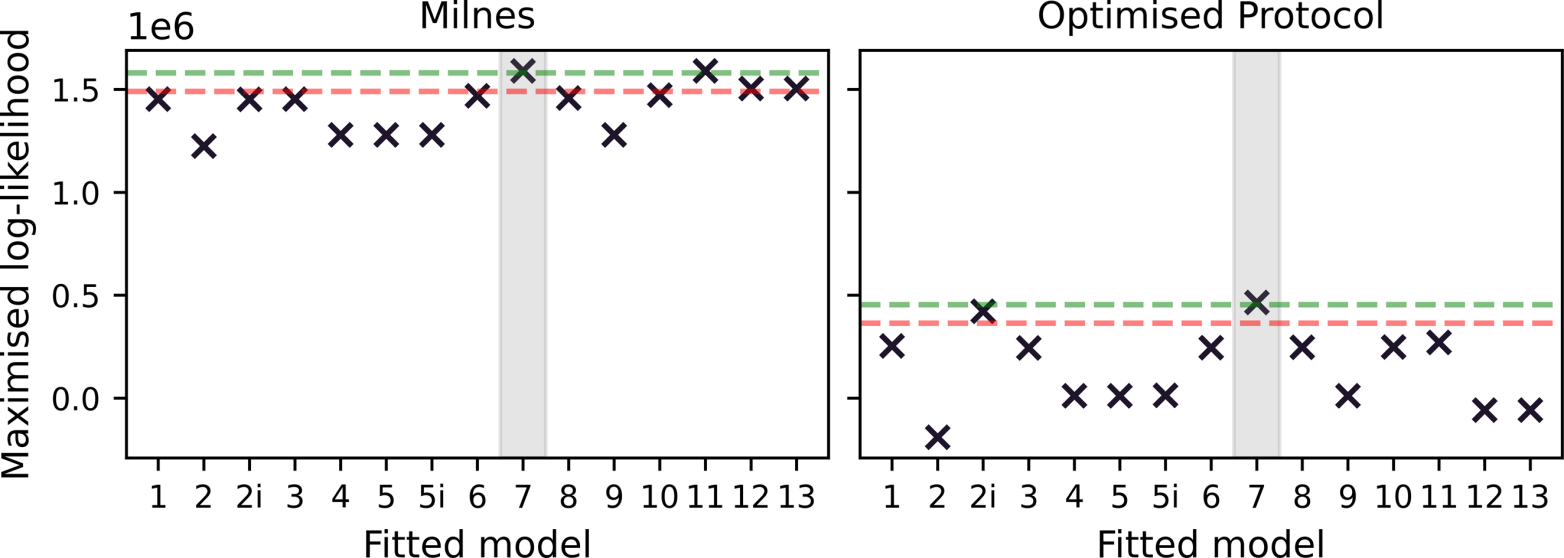
Maximized log-likelihoods for model fits to synthetic data generated under the modified Milnes protocol (left) and an optimized protocol (right). This is for the discrepant hERG model case; the data-generating hERG model is the Lu model, while the Lei 37°C is used for model fitting. In each plot, we include a green dashed line at $10^{4}$ below the largest maximized log-likelihood and a red dashed line at $10^{5}$ below the largest maximized log-likelihood. We also shade, in grey, around the data-generating binding model.

We can then repeat the procedure described in §3*a*–*d*, but this time using the Lu hERG model when generating any synthetic data (Model 7 is still used as the data-generating drug-binding model). In figure 8, we plot the log-likelihoods obtained using the Lu model as the true data-generating hERG model but (incorrectly) assuming that the 37°C Lei model is the correct hERG model when fitting the drug-binding models to the data.

We appear to get quite similar results to the non-discrepant case, with the optimized protocol once again spreading out the values of the log-likelihoods and pointing towards Model 7 as the data-generating model. However, as we see in §4, we find these methods are less effective for other data-generating drug-binding models when discrepancies are introduced. In the electronic supplementary material, we include equivalent plots to figures 2, 3, 5 and 6 in electronic supplementary material, figs. S3–S6, respectively. In electronic supplementary material, fig. S7, we also include a plot comparing the model parameters fitted to the Milnes data and the model parameters fitted to the optimized protocol data.

## 4. Applying the OED methodology

In §3, we saw how our methodology performed for synthetic data generated under one drug-binding model (Model 7) parameterized for one drug compound. We now repeat these methods across a range of models and drugs to demonstrate how the procedure performs in differing circumstances.

### (a) Verapamil: non-trapping dynamics

**Figure 9.**
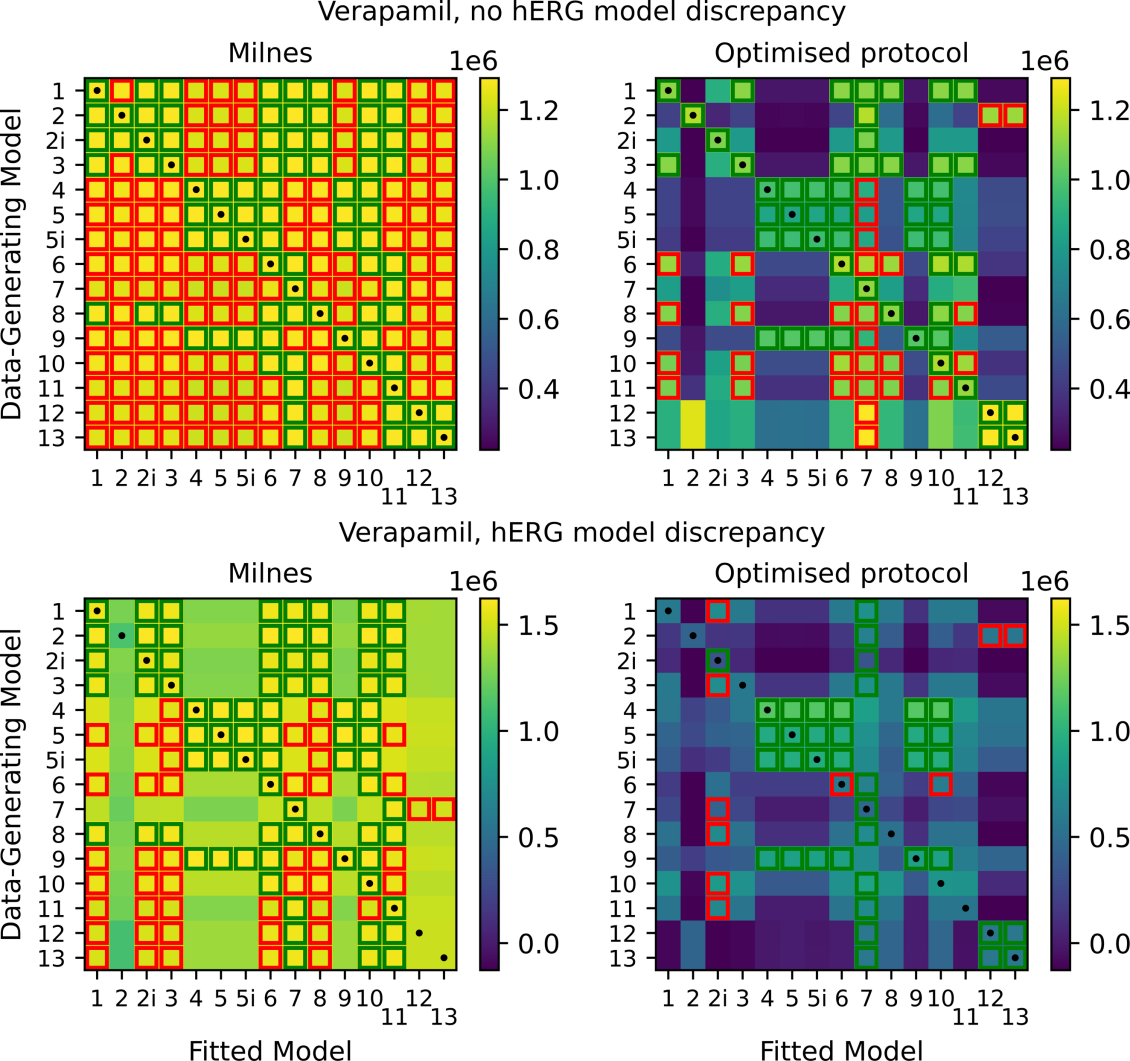
Top: heatmaps of maximized log-likelihoods for fitted models to Milnes (left) and optimized protocol (right) synthetic verapamil data. The fitted models within $10^{4}$ of the largest log-likelihood (very good fits) in each row are highlighted in green squares. The fitted models that are between $10^{4}$ and $10^{5}$ below the largest log-likelihood (reasonable fits) in each row are highlighted in red squares. Black dots are plotted down the diagonal of each heatmap where the fitted model corresponds to the data-generating model. Note that our optimal protocol results in a far bigger spread of maximized log-likelihoods. Bottom: equivalent heatmaps of maximized log-likelihoods with discrepancy between the data-generating hERG model and the hERG model used to fit the data. The introduced hERG model discrepancy makes identifying the true data-generating drug-binding model difficult.

**Figure 10.**
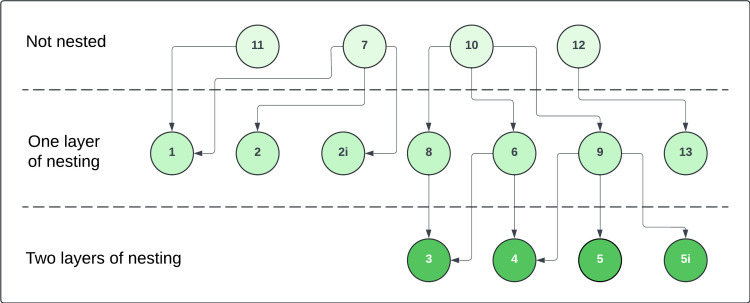
Graph illustrating nesting between drug-binding models. An arrow pointing from Model A to Model B indicates that Model B is nested within Model A.

We begin by considering verapamil again, but this time we generate synthetic data for each of the 15 drug-binding models (initially using the Lei model in figure 1*a* as our underlying hERG model). Starting with the previous fits to real verapamil data from [18] to generate the synthetic data, in the top half of figure 9, we plot heatmaps of the log-likelihoods obtained via the §3 methodology. This is for the case with no hERG model discrepancy. The *y*-axis represents which drug-binding model is used to generate the synthetic data, and we then get a corresponding log-likelihood for each fitted drug-binding model on the *x*-axis. We plot both the log-likelihoods obtained for the Milnes protocol data fits (top left), and for the optimized protocol data fits (top right). For each row, we highlight (with green squares) the fitted models within $10^{4}$ of the largest log-likelihood in that row. We then also highlight (with red squares) the fitted models that are within $10^{5}$ of the largest log-likelihood (that are not already highlighted in green). Black dots are plotted down the diagonal indicating where the fitted model corresponds to the data-generating model. The first notable observation from this plot is that the number of models within the $10^{4}$ and $10^{5}$ thresholds is significantly reduced in the optimized protocol case compared with the Milnes case. We also note that in both cases, the full diagonal is within the $10^{4}$ threshold. Note that there is a large amount of model nesting between drug-binding models, which we illustrate in figure 10. We consider some model, model A, to be nested within another model, model B, if by fixing one or more of the parameters in model B, we can reduce model B to model A. For this reason, we expect that (in this non-discrepant hERG case) if model A is nested within model B, and model A is the true data-generating model, then model B should also be able to fit the data at least equally well (perhaps even slightly better given higher complexity and more parameters), so the maximized log-likelihoods for Model A and B should be approximately equivalent. At the top of the tree diagram are the models that are not nested within any others; models 7, 10, 11 and 12. If we consider the heatmap in the top right-hand side of figure 9 corresponding to the optimized protocol, we see that for synthetic data generated under models 7, 10 and 11, the only fitted model with a log-likelihood within the $10^{4}$ threshold is the true data-generating model. Model 12, the other model with no nesting, only has one other model within this $10^{4}$ threshold (model 13). Considering the nested models in figure 10, we can explain some of the cases where multiple models sit within the $10^{4}$ threshold. In the optimized protocol case in the top right of figure 9, the $10^{4}$ threshold highlighted squares in the heatmap rows corresponding to models 2, 2i, 8 and 13 can all be explained by model nesting. On the other hand, data generated under models 1, 3, 4, 5, 5i, 6 and 9 all have at least one model within the $10^{4}$ threshold that cannot be explained by nesting.

We now switch to the discrepant hERG model case where synthetic data were generated using the Lu physiological hERG model, but we use the Lei model for fitting. In the bottom half of figure 9, we plot equivalent heatmaps to those at the top of the plot, but for the discrepant case. The purpose of considering this case is to address the more realistic scenario where we do not have a perfect model of hERG channel dynamics. We notice that the number of highlighted $10^{4}$ and $10^{5}$ threshold squares is once again significantly reduced in the optimized protocol case. However, in most cases, the best-fitting binding model is no longer the one that generated the data. As a result, the diagonal is highlighted significantly less than in the non-discrepant case, with only eight models (2i, 4, 5, 5i, 7, 9, 12 and 13) sitting within the $10^{4}$ threshold. Clearly, the hERG model discrepancy is causing issues with identifying the true binding mechanisms.

### (b) Bepridil: trapping dynamics

**Figure 11.**
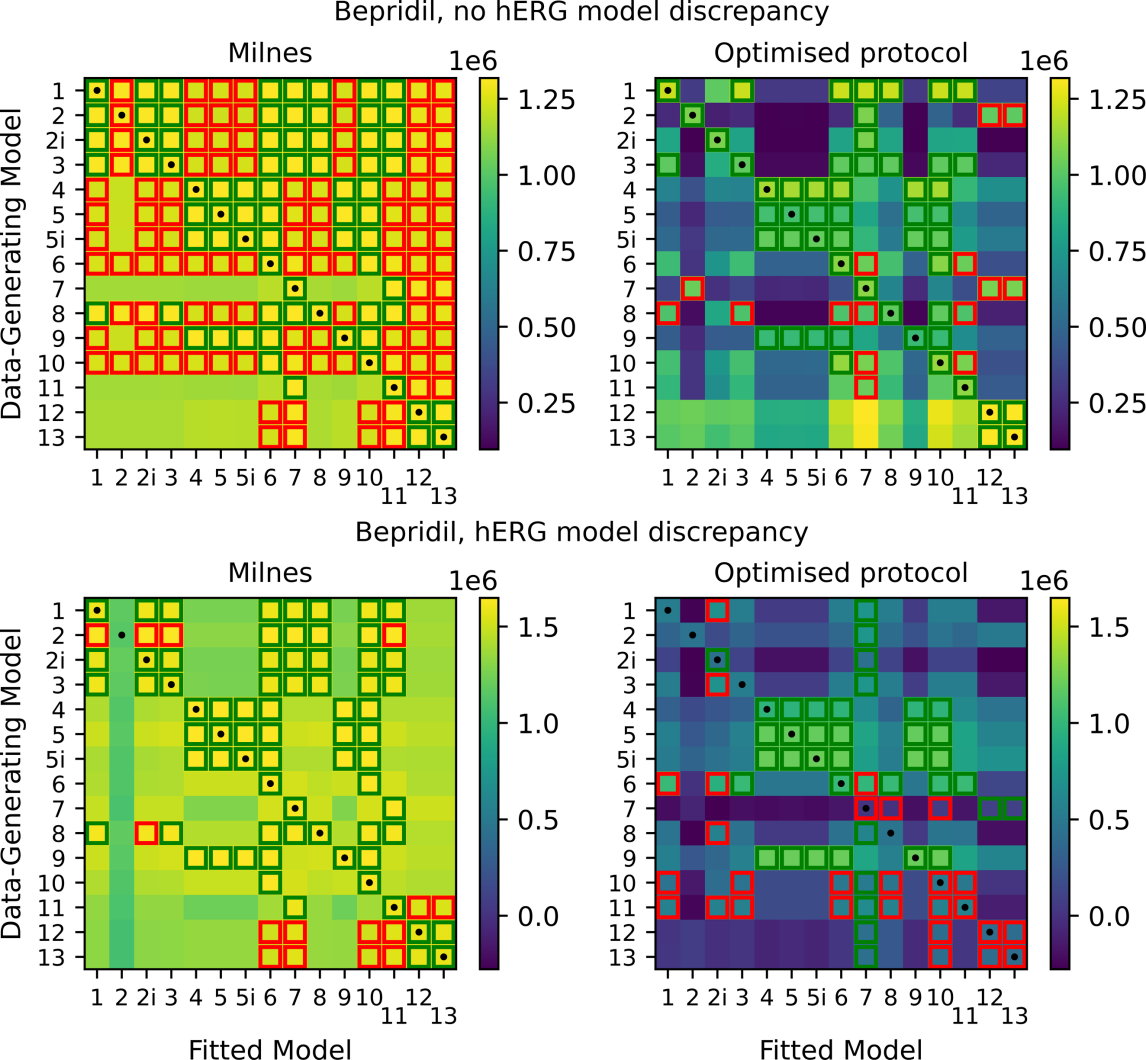
Top: heatmaps of maximized log-likelihoods for fitted models to Milnes and optimized protocol synthetic bepridil data. Bottom: equivalent heatmaps of maximized log-likelihoods with discrepancy between the data-generating hERG model and the hERG model used to fit the data. For discussion of highlighting see the caption of figure 9.

We can repeat the process described in the previous section for a different drug, this time with observed trapping behaviour [10,11]. In the electronic supplementary material, fig. S8, we include an example of model output for a drug with trapping behaviour, compared with one with no trapping behaviour. In the top half of figure 11, we plot the heatmaps of maximized log-likelihoods for the non-discrepant case as previously. The optimized protocol heatmap is very similar to that obtained with verapamil; the highlighted squares in the rows corresponding to models 1, 2, 2i, 3 and 8 are identical, while there are only a couple of differences for each of the other models. We can also again consider the discrepant hERG case, and we plot the heatmaps for this in the bottom half of figure 11. Again, this shows relatively similar results to those seen with verapamil; when we have discrepancy in the hERG model, it becomes difficult to determine the true data-generating drug-binding model. In the electronic supplementary material, fig. S9, we also include heatmaps for results obtained for chlorpromazine, a fast-binding drug with a suspected open-binding preference (over inactive-binding). These results appear similar to those obtained for verapamil and bepridil.

### (c) Dofetilide: slow-binding dynamics

**Figure 12.**
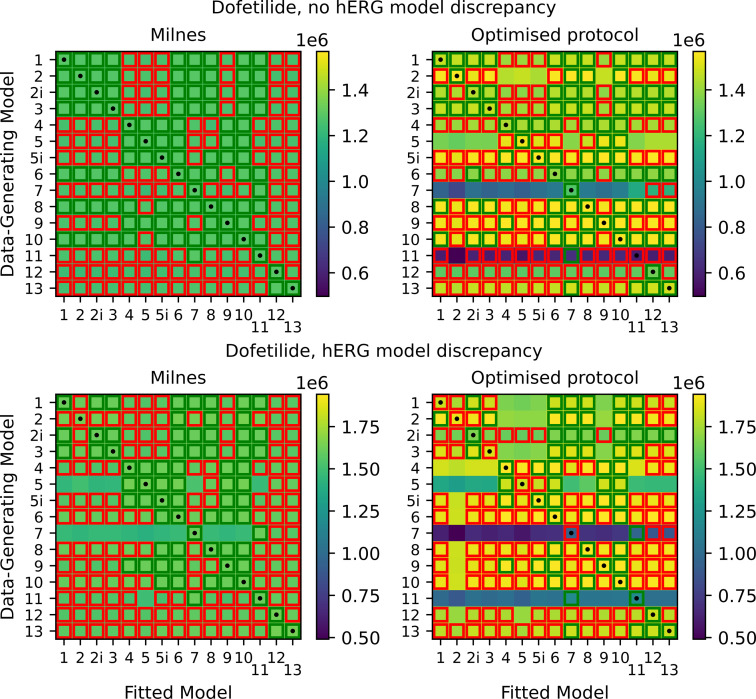
Top: heatmaps of maximized log-likelihoods for fitted models to Milnes and optimized protocol synthetic dofetilide data. Bottom: equivalent heatmaps of maximized log-likelihoods with discrepancy between the data-generating hERG model and the hERG model used to fit the data. For discussion of highlighting see the caption of figure 9.

Bepridil and verapamil both have relatively fast binding dynamics, so we also consider the slow-binding drug dofetilide [37]. In the electronic supplementary material, fig. S8, we include an example of model output for a drug with slow dynamics, compared with one with fast dynamics. Once again, we plot the heatmaps of maximized log-likelihoods as shown in figure 12, and we see that nearly all models can fit the Milnes data well. Unlike the fast dynamic drugs, with dofetilide, we get much less of a reduction in $10^{4}$ and $10^{5}$ threshold squares with the optimized protocol (in both the non-discrepant and discrepant cases). It is not obvious what causes this difference between fast and slow-binding drugs, and it may indicate that our median optimization objective function is less effective in the slow-binding case. We also include, in electronic supplementary material figs. S10–S13, heatmaps for each of the four considered drugs for the case where all model fitting is done assuming the Lu model is the true model of hERG dynamics (as opposed to the Lei model). These plots show similar overall results and indicate that the results obtained in the main text are not exclusive to the Lei hERG model.

## 5. Discussion

In this study, we have outlined a methodology for generating optimized voltage protocol designs to assist in distinguishing between models of drug-binding dynamics. By undertaking a synthetic data study, we have seen the potential benefits of this methodology when the true physiological model of hERG is known. Log-likelihoods of models fitted to data generated under our optimized protocols indicate a divergence in the quality of fits when compared with fits to data generated under a simple Milnes protocol. This suggests that this OED procedure could assist in establishing the true binding dynamics of a compound. The method was less effective when considering synthetic data emulating a slow-binding drug, dofetilide, when compared with drugs with faster dynamics such as verapamil and bepridil.

We also considered how discrepancy in the hERG model used to fit the data (compared with the data-generating hERG model) influenced the outcomes of our methodology. We found that when we used the Lu hERG model to generate synthetic data and the Lei 37°C hERG model to fit models to these data, log-likelihoods of model fits indicated that establishing the true data-generating drug-binding model became more difficult. This suggests that the underlying hERG model does play an important role when fitting drug-binding models to data and stresses the importance of continuing to improve basic models of physiological channel behaviour. A well-calibrated model of hERG current, that approximately matches the observed dynamics in real experimental data, would reduce the influence of model discrepancy on the outcomes of the OED procedure. In their 2019 paper, Clerx *et al.* [38] detail the benefits and limitations of several methods for calibrating models of ion channels. Our proposed methodology could perhaps be improved by considering fitting a hERG model to obtained control currents before fitting the drug-binding models (or fitting both the hERG and drug-binding models simultaneously).

We note that our results depend on the initial drug-binding model parameterizations for each of the three drug compounds, which come from model fits obtained by Lei *et al.* [18]. The quality of these model fits was quite variable from model to model and from compound to compound. This represents a limitation of a synthetic data study and motivates trialling the methodology on real data.

The methods used to fit the drug-binding models in this paper were developed to improve on those used by Lei *et al.* [18] and others [12,18,26]. While we have similarly fitted our models to the proportion of hERG in the open state, the log-likelihoods derived from the normal ratio PDF described above differ from the simple weighted sum-of-squares method used previously. This ratio likelihood fitting method provides a more realistic noise model for the data-generating process (in this synthetic scenario), avoiding problems associated with small control currents leading to large noise on the proportion open trace. In Lei *et al.* [18], the weighted sum-of-squares method required low currents at the start of each 10 s pulse to be cropped out to prevent noisy open proportion data from biasing the fit, our method allows us to fit the binding models to the full data sweep.

After some consideration and testing, the max-med optimality criterion was chosen over other possible alternatives such as maximizing the mean or the minimum of the pairwise sum-of-squares difference between model current traces. Using the mean or minimum, as opposed to the median, tended to result in one or two model pairwise differences biasing the objective function score and leaving many of the other current traces indistinguishable from each other. It would be useful to perform more rigorous comparisons between optimality criteria to see if there are scenarios where alternatives are more effective.

In the results above, we noted the presence of nesting between the binding models. This nesting suggests that perhaps reducing our optimization problem to consider only pairwise differences between the non-nested models (7, 10, 11 and 12) could be an alternative starting point given all other nested models are simplified versions of these. Another avenue to explore would be the use of multiple different protocols, each optimized to emphasize particular drug-binding properties perhaps, and examining the way different models need to compromise to fit data from each protocol has proved instructive in providing a lower bound on model discrepancy [39].

To conclude, the proposed OED methodology shows promise in determining the true binding dynamics, but care must be taken to ensure that we have a well-calibrated model of control hERG current if applied to a real data study.

## Data Availability

Code is freely available at: [40] under a BSD-3-clause open source licence, and a permanently archived version is available on Zenodo at [41]. Supplementary material is available online [42].
